# Rival Male Relatedness Does Not Affect Ejaculate Allocation as Predicted by Sperm Competition Theory

**DOI:** 10.1371/journal.pone.0002151

**Published:** 2008-05-14

**Authors:** Melissa L. Thomas, Leigh W. Simmons

**Affiliations:** Centre for Evolutionary Biology, School of Animal Biology, The University of Western Australia, Crawley, Australia; University of Edinburgh, United Kingdom

## Abstract

When females are sexually promiscuous, the intensity of sperm competition for males depends on how many partners females mate with. To maximize fitness, males should adjust their copulatory investment in relation to this intensity. However, fitness costs associated with sperm competition may not only depend on how many males a female has mated with, but also how related rival males are. According to theoretical predictions, males should adjust their copulatory investment in response to the relatedness of their male rival, and transfer more sperm to females that have first mated with a non-sibling male than females that have mated to a related male. Here, for the first time, we empirically test this theory using the Australian field cricket *Teleogryllus oceanicus*. We expose male crickets to sperm competition from either a full sibling or non-sibling male, by using both the presence of a rival male and the rival male's actual competing ejaculate as cues. Contrary to predictions, we find that males do not adjust ejaculates in response to the relatedness of their male rival. Instead, males with both full-sibling and non-sibling rivals allocate sperm of similar quality to females. This lack of kin biased behaviour is independent of any potentially confounding effect of strong competition between close relatives; kin biased behaviour was absent irrespective of whether males were raised in full sibling or mixed relatedness groups.

## Introduction

Kin selection theory predicts that preferential behaviour towards relatives will evolve if these behaviours increase the inclusive fitness of individuals indirectly through the reproduction of their relatives [1]. Kin selection theory can be applied to many situations, and is considered fundamental to explaining interactions between closely related conspecifics during reproduction. For example, a leading explanation for cooperative breeding in vertebrates and invertebrates is that individuals gain indirect fitness benefits by helping to rear relatives [e.g. 2], [3].

Recently, a theoretical model has been developed that examines kin biased behaviour during reproduction in a novel context; sperm competition between relatives [4]. Sperm competition is a widespread phenomenon that occurs when sperm from two or more males compete to fertilize the same set of eggs [5]. There is good evidence that relative sperm numbers can be important for sperm competitive success, so for males, an ability to assess the risk of sperm competition enables them to allocate sperm prudently [6], given the costs associated with producing sperm [e.g. 7], [8], [9]. Parker's [4] sperm competition model examines the effects of relatedness between competing males on sperm allocation at any particular mating, and predicts that males should exhibit a conditional shift in behavior, transferring less sperm if his male rival is a full sibling. Kin biased behaviour in this context allows males to maximize their inclusive fitness by allowing their brothers to sire more offspring, while conserving their own ejaculates for competition with non-sibling males.

Since the development of Parker's [4] sperm competition model, it has become apparent that strong competition between relatives can act as an opposing force to kin selection [10], [11]. This is particularly pertinent for species that have limited dispersal from their natal group. For example, in fig wasps, where males compete for mates within their natal fig, fighting between relatives can be so intense that it removes any kin selected benefit for reduced fighting among close relatives [10]. In this extreme case, competition is completely local, and any increase in reproduction of one relative comes at a cost to other relatives. Conversely, when competition is global, there is likely to be a negligible effect of competition between relatives [11]. For example, many species of birds disperse large distances after fledging from their natal nest, resulting in limited competition between related adults [e.g. 12]. Clearly, there are many situations that will fall between these two extremes, and empirical studies investigating kin selection theory need to consider the net effect of these two opposing forces.

In this study, we investigate for the first time Parker's [4] model on sperm competition between related males using the Australian field cricket *Teleogryllus oceanicus*. This cricket species has been widely used in sperm competition studies, and recent evidence shows that males do adjust their ejaculate expenditure in accordance with Parker's general sperm competition models [13], [14]. Moreover, there is evidence that this species has kin discriminatory abilities; female *T. oceanicus* display kin discrimination postcopulatory [15], and males possess chemosensory cues that reflect genetic relatedness [16].

To test Parker's [4] kin selection model, we measure ejaculate expenditure of males when they are exposed to sperm competition from either a full-sibling or non-sibling male competitor. We manipulate both the perceived risk and actual occurrence of sperm competition by exposing experimental males to the presence of a rival male (perceived risk), and his competing ejaculate (actual competition). Male *T. oceanicus* are known to be able to adjust their ejaculate expenditure in response to both of these sperm competition cues [13], [14]. We also manipulate the exposure of males to related conspecifics, to determine how variation in competition between relatives influences preferential behaviour towards relatives. We do this by raising crickets in either full sibling groups (high competition between close relatives), or mixed relatedness groups (low competition between close relatives). We expect that individuals raised in mixed-relatedness groups should be more likely to display preferential behaviour towards their relatives, than individuals raised in full sibling groups.

As a measure of ejaculate allocation we use the viability of sperm (proportion of live and dead sperm). We do not measure absolute sperm numbers because fertilization success of male *T. oceanicus* is not influenced by this trait [17]. In contrast, paternity success of *T. oceanicus* is determined by the proportion of live sperm in a male's ejaculate [18]. Moreover, male *T. oceanicus* have been shown to display phenotypic plasticity in the viability of their ejaculates in response to sperm competition risk and intensity [13], [14], but not in the numbers of sperm transferred at copulation [14]. One possible mechanism used by males to alter the viability of their sperm is to differentially invest in their seminal fluids. Seminal fluids may function to activate and/or nourish sperm during transportation and thereby influence the viability of sperm contained in the ejaculate. Seminal fluids are known to have important impacts on sperm quality [19], and there is good evidence to suggest that production of seminal fluids is costly [reviewed in 5]. We predict that males will invest more in their ejaculates, which will be reflected in the viability of sperm when competing with a non-sibling male.

## Methods

### Experimental animals

The parental generation of experimental crickets were the offspring derived from individuals collected from a banana plantation in Carnarvon, north-western Australia. We obtained experimental crickets by housing individual male crickets each with a non-sibling virgin female for one week. Mated females were then housed individually and allowed to oviposit on damp cotton wool. Newly hatched first generation nymphs were raised in 5 litre plastic containers in a constant temperature room, at 25°C with a 12:12 hr light dark cycle. Sexes were separated prior to the adult moult. After adult eclosion, crickets were isolated in individual boxes (7 cm×7 cm×5 cm) for 14±3 days before being used in experiments.

We conducted two separate experiments to test Parker's [4] sperm competition model. These experiments differed in the perceived intensity of competition between close relatives. In the first experiment, the first generation experimental crickets were raised in full sibling groups. Full sibling groups consisted of thirty newly hatched nymphs derived from the same singly mated female. In the second experiment, we reared a second generation of experimental crickets produced as above, but raised in mixed relatedness groups. Mixed relatedness groups consisted of fifteen newly hatched full sibling nymphs, and fifteen newly hatched non-sibling nymphs of mixed parentage, making a total of 30 individuals. In mixed relatedness groups, non-sibling nymphs were discernable from full sibling nymphs via a morphological marker, white eyes. We generated 27 families raised in full sibling groups in experiment 1, and 18 families raised in mixed relatedness groups in experiment 2.

### Mating trials

To test whether rival male relatedness influences ejaculate allocation of males, we assigned two full sibling males from each family to one of two rival male treatments; (1) a full sibling rival, or (2) a non-sibling conspecific rival. Rival males were of similar size and age to experimental males, and were all black eye morphs. We provided experimental males with information on the risk of sperm competition using both the perceived risk and actual occurrence of sperm competition. Experimental males were exposed to the perceived risk of sperm competition by placing them together with rival males in a small plastic box (7 cm×7 cm×5 cm) for one hour per day, over three consecutive days. To differentiate between rival and experimental males during this period, we clipped one of the rival male's wings. We exposed experimental males to actual sperm competition by mating rival males with the focal female. In crickets, sperm is transferred to females in a spermatophore, a discreet vessel containing sperm that remains attached outside the female's body following mating. To ensure that focal females did not remove spermatophores before sperm was transferred, we left rival males to guard them for forty minutes following copulation.

Before mating experimental males to the focal female, they were mated to a random unmated female and the spermatophore discarded. After this initial non-experimental mating, males were placed immediately with the focal female where they subsequently produced a fresh spermatophore. This new spermatophore therefore reflected a male's ejaculate investment in the focal female. Upon mating with the focal female, the spermatophore was immediately removed, and ejaculate quality measured.

Sperm viability was analysed using methods from García-González & Simmons [18]. In brief, we ruptured the spermatophore in 20 µL of Beadle saline (128.3 mM NaCl, 4.7 mM KCl, 23 mMCaCl2). We then mixed 5 µL of this sperm solution with 5 µL of a 1∶50 diluted 1 mM SYBR-14 solution (stains live sperm green) and kept the sample in the dark for ten minutes. Following this incubation period, we added 2 µL of 2.4 mM propidium iodide to the solution (stains dead sperm red) and left the solution for a further 10 minutes in the dark. Under a fluorescence microscope at 200 X magnification we scored five hundred sperm per sample to obtain proportions of live and dead sperm. Sperm viability was a measure of the proportion of these 500 sperm that were alive (stained green). Sperm counts were made blind to the experimental treatment.

We used two statistical approaches in analysing our data. In the first we used two-factor ANOVAs to look at the separate effects of family and relatedness of sperm competition rival on sperm viability. In the second, we increased our statistical power by ignoring potential differences between families from which our subjects were drawn, and using paired t-tests to contrast the viability of sperm from full siblings competing with a brother or an unrelated male. Means are presented ±1SE. Effect sizes were calculated using Cohen's *d* statistic [20], [21].

## Results

**Figure 1:**
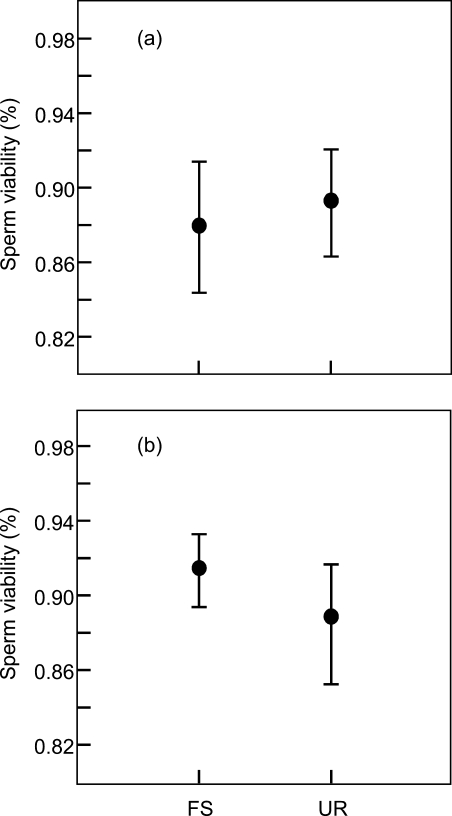
Reproductive response of males when competing with either a full sibling (FS) or non-sibling (NS) male rival; (a) males raised in full sibling groups (N = 27,) and (b) males raised in mixed relatedness groups (N = 18). Means±S.E are illustrated.

In contrast to theoretical predictions, male *T. oceanicus* did not alter expenditure on their ejaculates in response to rival male relatedness. We found that males with full sibling sperm competition rivals produced ejaculates containing sperm of similar viability as did males with non-sibling sperm competition rivals (Table 1, Figure 1). This result was obtained in our first experiment when experimental males were raised in full sibling groups, and in our second experiment when experimental males were raised in mixed relatedness groups (Table 1, Figure 1). Similar conclusions were drawn when analysing the data with the alternative approach of using paired t-tests (full sibling groups, t = −0.301, d.f. = 26, p = 0.766; mixed relatedness groups, t = 0.730, d.f. = 17, p = 0.475).

**Table 1:** Two-way ANOVA for the effect of rival male relatedness (full sibling or non-sibling) and family, on the viability of ejaculates allocated to females.

	d.f.	SS	F	p
**(a) full sibling group**				
Relatedness	1	0.001	0.094	0.762
Family	26	0.219	1.061	0.440
Error	26	0.206		
**(b) mixed relatedness group**				
Relatedness	1	0.002	0.533	0.473
Family	17	0.047	0.705	0.748
Error	17	0.066		

Experimental males were raised in either (a) full sibling groups or (b) mixed relatedness groups.

The effect sizes (Cohen's *d*, {95%CI}) of relatedness on sperm viability were 0.04 {−0.10, 0.19} for full sibling groups and 0.12 {−0.10, 0.34} for mixed relatedness groups. These effect sizes contrast strongly with those calculated using data from Thomas and Simmons [14], for the effect of female mating status on male investment in sperm viability (0.71 {0.59, 0.83}).

## Discussion

Our results show that male *T. oceanicus* do not alter the viability of their sperm in response to the local relatedness of rival males; males transfer sperm of similar viability irrespective of whether they are competing with a full sibling or non-sibling male. This result is contrary to a theoretical model by Parker [4] which predicts that males should invest more in their ejaculate when females have first mated with a non-sibling male than when females have mated with a related male. This lack of kin-biased behaviour by males is unlikely to be related to any constraints on the phenotypic plasticity of sperm viability. Previous research using *T. oceanicus* has shown that males are sensitive to the fitness costs and benefits associated with sperm competition; males do exhibit short-term phenotypic plasticity in sperm viability in response to female mating status and the presence of rival males [13]. In addition, our absolute sperm viability estimates are consistent with male sperm investment in singly mated females [14].

Parker's [4] original model used sperm number as the currency of male ejaculate expenditure. However, it is becoming increasingly clear that sperm numbers are not the only aspect of male expenditure that will contribute to fertilization success [22]. Several recent studies have revealed male strategic adjustments in the quality of sperm; human males produce ejaculates with faster swimming sperm when exposed to cues of sperm competition [23], and male jungle fowl transfer ejaculates with faster swimming sperm when mated with high quality females [24]. Variations in seminal fluid components that influence sperm quality offer the most parsimonious explanation for these results. Importantly, a recent theoretical analysis that follows Parker's logic, has shown that males should also adjust non-sperm components of the ejaculate in relation to sperm competition risk and intensity [25]. Although *T. oceanicus* appear to adjust their ejaculate quality to the risk and intensity of sperm competition (Thomas & Simmons 2007; Simmons et al. 2007), they do not appear to do so in relation to the relatedness of rival males.

In insects, the most common type of label used for recognition of kin are chemicals. In many cases cuticular hydrocarbons, the waxy substances found on the exoskeleton of most insect species, have been implicated [see 26 for review]. Quantitative genetic analysis of cuticular hydrocarbons in *T. oceanicus* indicates that males have sufficient phenotypic and genetic variation in this trait to distinguish kin from non-kin [16]. So the lack of kin biased behaviour displayed by male *T. oceanicus* in our study is unlikely to be a result of any mechanistic constraints imposed by the production or expression of a kin label. However, kin discrimination is not just based on the expression of a kin label; individuals must also be able to perceive and then subsequently act on this phenotypic cue [27], [28].

The perception of kin recognition cues are based primarily on two mechanisms: discrimination by association and phenotype matching [29], [30]. Discrimination by association involves identifying individuals as kin through previous direct familiarity with each of them [30]. In this system, discrimination would most probably be acquired through a learning process [reviewed in 31]. Learning and memory of chemical cues and scents has been well demonstrated in crickets [32], [33], and in the cricket *Gryllus bimaculatus*, the recognition of kin is greatly enhanced if individuals are allowed to learn the characteristics of non-sibling conspecifics [34]. In our study we found that individuals raised in both full sibling and mixed relatedness groups displayed the same lack of kin-biased behaviour, suggesting that male *T. oceanicus* do not discriminate kin by association, and that experience with non-sibling conspecific odours does not enhance kin discrimination in this species, at least in the context of strategic ejaculation. The other main mechanism used for the perception of kin is phenotype matching. Phenotype matching is a process by which one individual assesses how well the phenotypic cue of another individual matches their own [reviewed in 26], [35]. Phenotype matching appears to be used as a discriminatory mechanism by female crickets, *Gryllodes sigillatus* [36], and the family specificity in cuticular hydrocarbons found in *T. oceanicus* indicates a putative mechanism of chemosensory self-recognition [16]. However, it should be noted that the actual occurrence of kin recognition based on matching with one's own phenotypic cues is widely debated, and new theory indicates that genetic kin recognition is inherently unstable [37], [38].

There is evidence to suggest that female *T. oceanicus* show differential behaviour based on kinship, fertilizing their eggs with sperm from non-sibling males rather than full-siblings [15]. If males possess similar kin discriminative abilities, they do not appear to respond to this information, at least in the context of Parker's [4] sperm competition model. Males may not display kin biased behaviour in this context, if there are few fitness benefits associated with discrimination. Our study investigates one such scenario; when there is strong competition between relatives. Competition between relatives is thought to oppose the evolution of altruistic behaviour, because altruism towards a related individual is less advantageous if their increased fitness comes at a cost to other relatives [11]. However, we found that irrespective of whether males were raised in full sibling (strong competition between relatives) or mixed relatedness groups (weaker competition between relatives), males invested sperm of similar viability to females when competing against a sibling or non-sibling male.

Whatever the reasons for a lack of kin-biased behaviour displayed by male *T. oceanicus*, it is certainly not the first species where kin biased behaviour has been investigated, but not found [38], [39]. In fact, an absence of kin biased behaviour appears to be a relatively common phenomenon, particularly in non-social insects [39]. An analysis of the presence/absence of kin recognition across this and other taxa would be interesting, and may provide insights into the underlying selection processes that shape this behaviour.
